# The role of Asprosin in patients with dilated cardiomyopathy

**DOI:** 10.1186/s12872-020-01680-1

**Published:** 2020-09-07

**Authors:** Ming-Shien Wen, Chao-Yung Wang, Jih-Kai Yeh, Chun-Chi Chen, Ming-Lung Tsai, Ming-Yun Ho, Kuo-Chun Hung, I-Chang Hsieh

**Affiliations:** 1 Department of Cardiology, Chang Gung Memorial Hospital, and Chang Gung University College of Medicine, 5 Fu-Hsing Street, Taoyuan, 333 Taiwan; 2 Institute of Cellular and System Medicine, National Health Research Institutes, Zhunan, 350 Taiwan

**Keywords:** Asprosin, Dilated cardiomyopathy, Heart failure, Hypoxia, Obesity

## Abstract

**Background:**

Asprosin is a novel fasting glucogenic adipokine discovered in 2016. Asprosin induces rapid glucose releases from the liver. However, its molecular mechanisms and function are still unclear. Adaptation of energy substrates from fatty acid to glucose is recently considered a novel therapeutic target in heart failure treatment. We hypothesized that the asprosin is able to modulate cardiac mitochondrial functions and has important prognostic implications in dilated cardiomyopathy (DCM) patients.

**Methods:**

We prospectively enrolled 50 patients (86% male, mean age 55 ± 13 years) with DCM and followed their 5-year major adverse cardiovascular events from 2012 to 2017. Comparing with healthy individuals, DCM patients had higher asprosin levels (191.2 versus 79.7 ng/mL, *P* < 0.01).

**Results:**

During the 5-year follow-up in the study cohort, 16 (32.0%) patients experienced adverse cardiovascular events. Patients with lower asprosin levels (< 210 ng/mL) were associated with increased risks of adverse clinical outcomes with a hazard ratio of 7.94 (95% CI 1.88–33.50, *P* = 0.005) when compared patients with higher asprosin levels (≥ 210 ng/mL). Using cardiomyoblasts as a cellular model, we showed that asprosin prevented hypoxia-induced cell death and enhanced mitochondrial respiration and proton leak under hypoxia.

**Conclusions:**

In patients with DCM, elevated plasma asprosin levels are associated with less adverse cardiovascular events in five years. The underlying protective mechanisms of asprosin may be linked to its functions relating to enhanced mitochondrial respiration under hypoxia.

## Introduction

Heart failure (HF) is a growing global public health problem, particularly in the elderly population, affecting more than 26 million patients worldwide [1]. The health care expenditure attributed to heart failure in Europe and North America reaches around 1–2% recently [2, 3].

Despite encouraging advances in heart failure medications and device therapy, mortality and readmission rates in HF patients are still around 15–30% over the past 15 years [4]. The development of new therapeutics to avoid HF readmission are the current major target of many studies. Therefore, it is imperative to explore a novel mechanism to prevent heart failure or to develop an efficient target for improving heart function [5].

Most current HF treatments emphasize reducing myocardial workload and energy consumption, such as alleviating preload or afterload, reducing heart rate, and modulating neurohormonal activation [6]. Over the last decades, studies have focused on cardiac energy metabolism. This leads to the discovery of several novel compounds to balance the energy expenditure mismatching of HF patients [7, 8]. Previous studies have found that there are significantly decreased mitochondria energy generation and transfer capacity in the cardiomyocytes of HF patients [9–11]. The bioenergetics impairment in the failing heart is reflected by underlying mitochondrial abnormalities, such as impaired electron transport chain activity [12], increased formation of reactive oxygen species [13], shifted metabolic substrate utilization [14, 15], and altered ion homeostasis [16].

Asprosin is a novel fasting-induced glucogenic adipokine [17]. It is a C-terminal cleavage product of profibrillin. Asprosin mainly secrets from white adipose tissues into circulation [18]. Asprosin stimulates glucose releases from hepatic cells through activation of the G protein-cyclic AMP-protein kinase A pathway. The secretion of asprosin displays a circadian oscillation and is influenced by the dietary supplements [19]. Overnight fasting results in increases in asprosin concentrations while feeding decreases asprosin concentrations. Furthermore, elevated plasma asprosin concentrations are found to be associated with serum insulin levels and insulin resistance in humans and mice [20]. In opposition, patients with truncating mutation of FBN1 coding profibrillin have lower plasma asprosin and insulin levels than unaffected people [21]. Moreover, higher asprosin concentrations before bariatric surgery were associated with the weight reduction magnitude at 6 months after surgery [22].

Given the switching of energy source from fatty acid toward glycolytic pathways in the mitochondria of failing hearts, rapid and precise regulation of plasma glucose by glucogenic adipokines might be an important mechanism to fulfill the energy requirement of cardiomyocytes, especially in response to hemodynamic stress. Based on this energy adaptation mechanism, we hypothesized that asprosin can modulate the clinical outcomes in patients with dilated cardiomyopathy (DCM) and affect mitochondrial functions in cardiomyocytes.

## Methods

### Study design and population

We prospectively enrolled patients presented with heart failure and have a diagnosis of dilated cardiomyopathy at the Chang Gung Memorial Hospital [23]. The diagnostic criteria of dilated cardiomyopathy included (1) typical heart failure symptoms, such as shortness of breath, pulmonary or peripheral edema and elevated jugular venous pressure; (2) transthoracic echocardiography evaluation showed left ventricular or biventricular systolic dysfunction and dilatation by the European Society of Cardiology guideline [24]. The left ventricular ejection fraction was below 35%, which was calculated using the modified Simpson’s biplane method with apical four-chamber view; (3) the absence of hypertension, valvular heart disease, diabetes mellitus, heavy alcohol consumption, cardiotoxicity drug exposure and systemic inflammatory diseases in a series of diagnostic workup, (4) no coronary artery abnormalities confirmed by cardiac catheterization. The plasma brain natriuretic peptide (BNP) was also measured. Health participants without a previous history of heart diseases with normal left ventricular ejection fraction by echocardiography and normal exercise treadmill tests served as the normal control subjects.

The pre-specified composite endpoints include all-cause mortality, heart failure re-hospitalization, cardiac transplantation, stroke, new-onset atrial fibrillation, ventricular tachycardia, and implantable cardioverter-defibrillator. Heart failure re-hospitalization was defined as hospital readmission with heart failure treatment, which requiring diuretics, vasodilators, or inotropic agents. When patients had multiple events, the time to the first event was counted as the censored outcome. All patients provided written informed consent. These studies complied with the declaration of Helsinki, and the study protocol was approved by the institutional review board of the Chang Gung Memorial Hospital.

### Serum samples and measurement of asprosin

Venous blood was drawn from patients and normal subjects after 8 h of fasting and plasmas were stored at − 80 °C until analysis. For the designing and characterization of the endogenous asprosin sandwich ELISA, a mouse monoclonal anti-asprosin antibody against asprosin amino acids (human profibrillin amino acids 2832–2871) was used as the capture antibody and a goat anti-asprosin polyclonal antibody against asprosin amino acids 6–19 (human profibrillin amino acids 2737–2750) by Abnova was used as the detection antibody. An anti-goat secondary antibody linked to HRP was used to generate a signal. Serial dilution assay and spike/recovery test against human asprosin protein as positive control were used to establish the accuracy of the ELISA [22].

### Cell culture and protein analysis

The H9c2 cell line was from American Tissue Type Collection (catalog # CRL-1446) and cultured in DMEM medium supplemented with 1.5 g/L sodium bicarbonate, 10% fetal bovine serum, 100 U/ml penicillin and 100 μg/ml streptomycin at 37 °C in a humidified atmosphere of 5% CO2. Cells were fed every 2 days and sub-cultured when reaching 70–80% confluence to prevent the changes of cellular phenotype. In order to establish the hypoxic condition, H9c2 cells were transferred to an airtight Plexiglas hypoxic chamber in a glucose-free medium. The hypoxic condition (< 1%) was achieved by flushing the chamber with 5% CO_2_ and 95% N_2_. The hypoxia exposure was confirmed with O_2_ concentration monitoring and elevation of HIF-1α protein abundance with Western blotting. Cell viability was examined with trypan blue exclusion tests. Specifically, 0.4% trypan blue in buffered isotonic salt solution was used to stain the H9c2 cells. The blue staining cells were considered non-viable.

### Mitochondrial function measurements

Measurement of cardiomyoblasts respiratory was performed using the Seahorse XF24 analyzer (Seahorse Bioscience Inc.). H9c2 cells were plated at a density of 20,000 cells/well on the XF24 plate. The cells were treated with 2.5 μg/mL asprosin or vehicles for 24 h and then exposed with or without 3% H_2_O_2_ for 4 h. Prior to the respiration assay, cells were rinsed and cultured in DMEM running medium (8.3 g/L DMEM (Sigma), 200 mM GlutaMax-1 (Invitrogen), 100 mM sodium pyruvate (Sigma), 25 mM D-glucose (Sigma), 63.3 mM NaCl (Sigma), and phenol red (Sigma), adjust pH to 7.4 with NaOH). Oxygen consumption was measured under basal conditions, in the presence of the mitochondrial inhibitors oligomycin (1 μM) or mitochondrial uncoupler FCCP (4 μM), or antimycin A/rotenone (0.5 μM) to analyze maximal oxidative capacity and proton leak.

### Statistical analyses

Continuous variables with normal distribution were described as mean ± standard deviation and Student t-tests were used for comparison. If not normally distributed, variables were described as median with interquartile range and compared with the Mann-Whitney test. A cutoff value of elevated asprosin (≥ 210 ng/mL) level was determined according to the median level of asprosin in the study cohort. The association of asprosin level and time to adverse cardiac outcomes was assessed with multivariable Cox proportional hazard analysis. Major adverse event-free survival curves were generated from the Kaplan–Meier survival analysis. Statistical analyses were performed using SPSS version 22 (IBM) and Prism 8 (GraphPad Software, LLC). *P* values < 0.05 were considered statistically significant.

## Result

### Subject characteristics

**Fig. 1:**
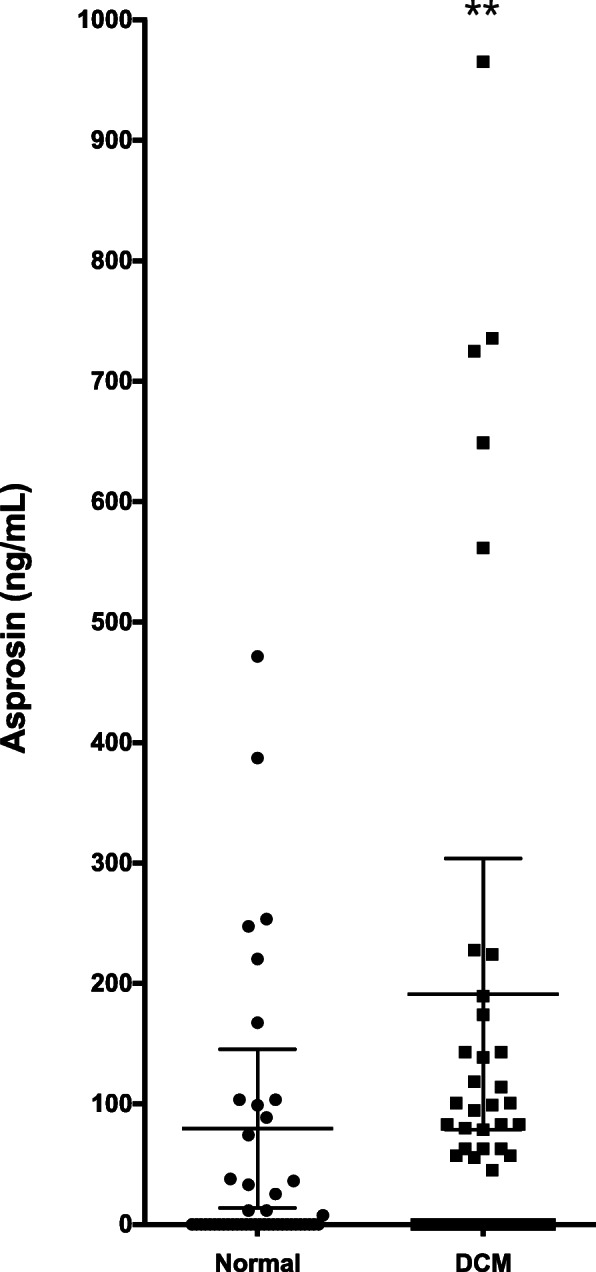
Asprosin levels in patients with dilated cardiomyopathy. Scatter plot (mean with 95% confidence interval) of serum asprosin levels. Comparison of serum samples from patient with dilated cardiomyopathy (*n* = 50) and normal control subjects (*n* = 50). **P < 0.01 vs normal control, by Mann-Whitney test. Asprosin levels > 1000 μg/ml (1754–65,053) which belonged to 5 dilated cardiomyopathy patients were not plotted in the graph

Baseline characteristics for patients with DCM cohort are presented in Table 1. Patients with DCM had a mean age of 55 ± 10 years and a mean BNP concentration of 1073 ± 1199 pg/mL; 14.0% female and 30.0% were in New York Heart Association (NYHA) class III or IV. Mean asprosin level was 191.2 ng/mL (*n* = 50; 95% confidence interval (CI) of mean 78.8–303.6 ng/mL) in patients with DCM and 79.7 ng/mL in normal control subjects (*n* = 50; 95% CI of mean 13.9–135.5 ng/mL; age, gender, and body mass index (BMI) matched). Patients with DCM have significantly higher asprosin levels when compared with the normal subjects (Fig. 1). Patients were allocated into two groups according to the median levels of asprosin. Patients with lower or higher asprosin levels exhibited no differences in age, gender, body mass index, left ventricular ejection fraction, B-type natriuretic peptide (BNP), and other clinical parameters (Table 1). The median length of follow-up was 62 months. During the follow-up period, 2 patients died, 12 readmitted with heart failure, and 2 suffered strokes.

**Table 1:** Baseline Characteristics

Variable	All patients (*n* = 50)	Asprosin		*P* value
		Low group (*n* = 25)	High group (*n* = 25)	
Age, y	55 ± 10 (35–78)	55 ± 11 (35–78)	54 ± 9 (36–71)	0.98
Female gender, *n* (%)	7 (14.0%)	4 (16.0%)	3 (12.0%)	> 0.99
Body mass index, kg/m^2^	25.8 ± 4.3	25.2 ± 4.6	26.4 ± 4.1	0.44
Hypertension, *n* (%)	14 (28.0%)	6 (24.0%)	8 (32.0%)	0.75
Diabetes mellitus, *n* (%)	12 (24.0%)	6 (24.0%)	6 (24.0%)	> 0.99
Systolic blood pressure, mmHg	115 ± 21	112 ± 22	118 ± 19	0.25
Heart rate, bpm	80 ± 16	82 ± 16	79 ± 18	0.47
NYHA class III or IV, *n* (%)	15 (30.0%)	7 (28.0%)	8 (32.0%)	> 0.99
COPD, *n* (%)	0 (0.0%)	0 (0.0%)	0 (0.0%)	> 0.99
Atrial fibrillation, *n* (%)	17 (34.0%)	8 (32.0%)	9 (36.0%)	> 0.99
LV mass index, g/m^2^	344.9 ± 104.6	358.0 ± 110.7	331.8 ± 98.6	0.46
LV end-diastolic volume index, mL/m^2^	248.9 ± 74.9	248.1 ± 81.3	249.7 ± 69.7	0.99
LV ejection fraction, %	27.1 ± 9.9	26.5 ± 10.7	27.7 ± 9.2	0.63
Mitral *E*/*A* ratio	1.4 ± 0.9	1.4 ± 0.7	1.4 ± 1.1	0.85
ACEI/ARB, *n* (%)	44 (88.0%)	23 (92.0%)	21 (84.0%)	0.67
Beta-blockers, *n* (%)	42 (84.0%)	21 (84.0%)	21 (84.0%)	> 0.99
Spironolactone, *n* (%)	30 (60.0%)	15 (60.0%)	15 (60.0%)	> 0.99
Loop diuretics, *n* (%)	36 (72.0%)	19 (76.0%)	17 (68.0%)	0.75
Digoxin, *n* (%)	21 (42.0%)	10 (40.0%)	11 (44.0%)	> 0.99
BNP, pg/mL	1073 ± 1199	1208 ± 1370	955 ± 1046	0.74
eGFR, mL/min/1.73 m^2^	75.4 ± 24.5	77.1 ± 22.9	73.7 ± 26.3	0.73
Creatinine, mg/dL	1.5 ± 1.8	1.7 ± 2.4	1.2 ± 0.7	0.94
Cholesterol, mg/dL	156.4 ± 35.0	147.4 ± 31.3	165.5 ± 36.8	0.09
Sugar, mg/dL	103.8 ± 24.9	99.6 ± 16.7	108.2 ± 31.1	0.43
HbA1C, %	6.2 ± 1.1	6.1 ± 1.0	6.4 ± 1.3	0.54

*ACEI* angiotensin-converting-enzyme inhibitor, *ARB* angiotensin receptor blocker, *BNP* B-type natriuretic peptide, *COPD* chronic obstructive pulmonary disease, *eGFR* estimated glomerulus filtration rate, *HbA1C* glycohemoglobin, *LV* left ventricle, *NYHA* New York Heart Association

### Asprosin levels and adverse cardiovascular events in DCM patients

**Fig. 2:**
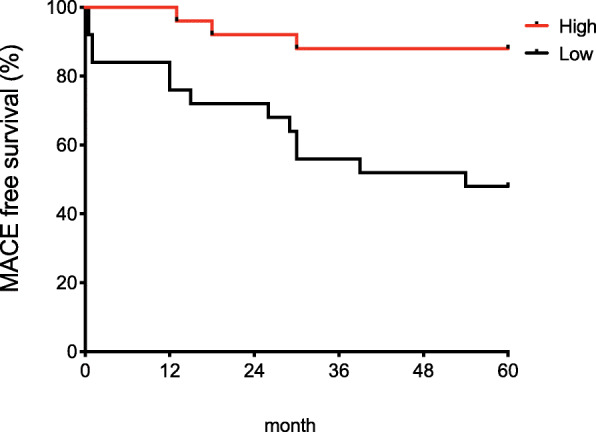
Kaplan-Meier estimates of 5-year adverse cardiovascular events (MACE)-free survival rates in patients with dilated cardiomyopathy. Patients with dilated cardiomyopathy (*n* = 50) stratified per optimal cutoff for serum asprosin as follows: Low asprosin (< 210 μg/ml) or High asprosin (≥ 210 μg/ml). The log-rank test was used to compare event-free survival trends (*P* = 0.0023)

In our study cohort, 16 (32.0%) patients experienced an adverse event of death, heart failure hospitalization, and strokes over the 5-year follow-up. When divided into a dichotomous variable of asprosin with a cut point of 210 ng/mL, elevated asprosin was a predictor of decreased risk of 5-year adverse events (Fig. 2). Low asprosin levels showed a strong effect on adverse events in univariate Cox proportional hazard analysis with a hazard ratio of 7.94 (95% CI 1.88–33.50; *P* = 0.005) for the adverse long-term event outcomes (Table 2). Age, LV ejection fraction, and cholesterol were also significantly associated with future heart failure-related events (Table 2). Multivariate Cox proportional hazards analysis including the age, LV ejection fraction, and cholesterol showed that the lower asprosin level was an independent predictor of future heart failure-related events (Table 3).

**Table 2:** Cox proportional hazard analyses of adverse long-term event outcomes

Variable	Univariate analysis	
	HR (95% CI)	*P* value
Asprosin (< 120 vs ≥120 ng/mL)	7.94 (1.88–33.50)	0.005
Age, y	0.93 (0.87–1.00)	0.04
Female gender, *n* (%)	0.83 (0.14–4.8)	0.83
Body mass index, kg/m^2^	0.09 (−0.06–0.23)	0.24
Hypertension, *n* (%)	1.26 (0.34–4.65)	0.73
Diabetes mellitus, *n* (%)	0.08 (−1.30–1.46)	0.91
NYHA class III or IV, *n* (%)	0.70 (0.18–2.67)	0.60
Atrial fibrillation, *n* (%)	1.26 (0.36–4.34)	0.72
LV mass index, g/m^2^	1.00 (0.99–1.01)	0.793
LV end-diastolic volume index, mL/m^2^	1.00 (0.99–1.01)	0.28
LV ejection fraction, %	0.92 (0.84–0.99)	0.03
Mitral *E*/*A* ratio	1.33 (0.46–3.86)	0.60
BNP, pg/mL	1.00 (0.99–1.00)	0.20
eGFR, mL/min/1.73 m^2^	1.00 (0.98–1.03)	0.79
Creatinine, mg/dL	0.66 (0.25–1.76)	0.40
Cholesterol, mg/dL	0.97 (0.95–0.99)	0.007
Sugar, mg/dL	0.98 (0.95–1.02)	0.32

*BNP* B-type natriuretic peptide, *eGFR* estimated glomerulus filtration rate, *LVEF* left ventricle ejection fraction, *NYHA* New York Heart Association

**Table 3:** Multivariate COX proportional hazards analysis to identify predictors of heart failure-related events using forced inclusion models

Variable	Model 1		Model 2		Model 3	
	HR (95% CI)	*P* value	HR (95% CI)	*P* value	HR (95% CI)	*P* value
Asprosin, (< 120 vs ≥120 ng/mL)	10.53 (2.18–50.81)	0.003	8.72 (1.90–39.97)	0.005	7.13 (1.51–33.69)	0.013
Age, y	0.92 (0.85–0.99)	0.026	–		–	
LVEF, %	–		0.91 (0.84–0.99)	0.036	–	
Cholesterol, mg/dL	–		–		0.97 (0.94–0.99)	0.02

*LVEF* left ventricle ejection fraction

The Kaplan-Meier survival analysis showed that heart failure-related events increased with the lower asprosin levels during the 60 months follow-up periods (Fig. 2).

### Treatment of asprosin significantly increased cell viability under hypoxic conditions

**Fig. 3:**
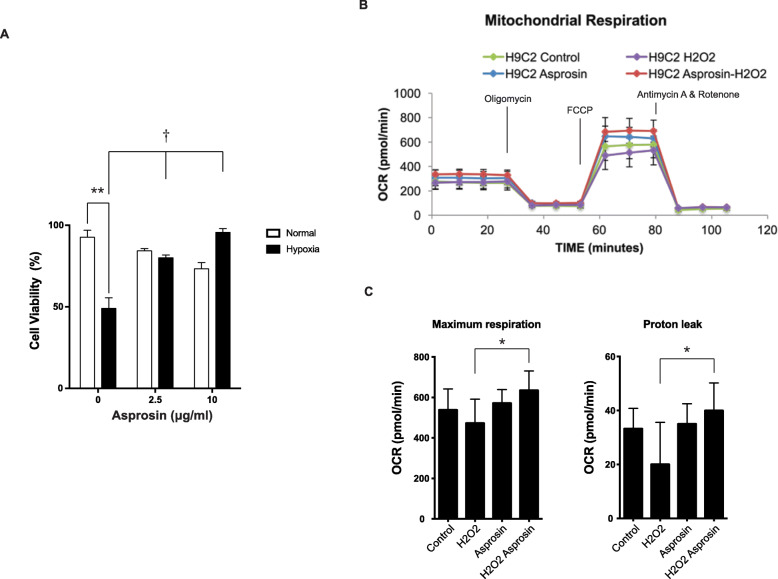
**a** Asprosin prevents hypoxia-induced cell death. Cardiomyoblast H9c2 cells were treated with different doses of asprosin for 24 h and exposed to hypoxia and normoxia conditions for 6 h. Cell viability was examined with a trypan blue exclusion test (*n* = 4 in each group). One-way analysis of variance with Greisser-Greenhouse correction and Holm-Sidak’s multiple comparisons were used to calculate the changes in cell viability. **P < 0.01, normoxia vs hypoxia without asprosin. †*P* < 0.05, asprosin 2.5 or 10 μg/ml vs 0 μg/ml. **b** Asprosin increases mitochondrial respiration and proton leak. Cardiomyoblasts H9c2 cells were treated with asprosin 2.5 μg/ml for 24 h and exposed to 3% H_2_O_2_ for 4 h (*n* = 4). Mitochondrial respiratory analysis of oxygen consumption rate. **c** Quantification of maximal respiration and proton leak. *P < 0.05, H_2_O_2_ vs H_2_O_2_ with Asprosin group by Mann-Whitney test

To explore the direct function of asprosin in cardiomyocytes, we treated cardiomyoblasts H9c2 cells with asprosin under hypoxia and normoxia conditions. After 6 h of hypoxia, we observed a marked decrease in cell viability without treatment of asprosin. Treatment of asprosin significantly increased cell viability under hypoxic conditions (Fig. 3a). Furthermore, cells treated with the higher concentration (10 μg/ml) of asprosin exhibited enhanced cell viability when compared with lower concentration asprosin (2.5 μg/mL).

### Asprosin increases mitochondrial respiration and proton leak

To further study the effects of asprosin on mitochondrial functions in cardiomyoblast, we exposed H9c2 cells with 2.5 μg/mL asprosin for 24 h and measured mitochondrial respiration with or without H_2_O_2_ (Fig. 3b). Asprosin significantly increased the mitochondrial maximum respiration and proton leak (Fig. 3c) under the exposure of H_2_O_2_ in H9c2 cardiomyoblasts, indicating that asprosin critically modulates mitochondrial function in H9c2 cardiomyoblast cells.

## Discussion

Our results demonstrated that asprosin, a novel glucogenic protein adipokine, exerts cardiac protective effects in patients with DCM. DCM patients with higher asprosin levels have less adverse cardiovascular events in 5 years. We showed that asprosin acts directly on cardiomyoblasts and enhances mitochondria respiration with proton leaks in responses to hypoxia.

Previous studies have shown that asprosin is recruited to the liver and enhances gluconeogenesis through its receptor OLFR734. Another study also shows that asprosin crosses the blood-brain barrier to activate appetite signaling. The asprosin receptor OLFR734 expresses in the olfactory epithelium, kidney, liver, muscles, adipose tissues, and testis. However, the asprosin action on cardiomyocytes is still unclear. Our data provided evidence that asprosin can act directly on cardiomyocytes and protect hypoxic injuries through mitochondrial respiration. Moreover, the asprosin abundances are affected by the dilated cardiomyopathy and associated with DCM outcomes. The OLFR734 is most abundant in the olfactory epithelium and adipose tissues [25]. It remained to be determined whether OLFR734 is the asprosin receptor in the cardiomyocytes and future study will be needed to investigate the signaling pathway of which asprosin exerts its protection from hypoxia in the heart and DCM patients.

DCM patients with higher asprosin levels in our cohort experienced fewer major cardiovascular events compared to patients with lower asprosin levels in five years follow up. Currently, the exact regulations of serum asprosin levels in humans are still unclear. Plasma asprosin exhibits an oscillatory circadian rhythm. Fasting, obesity, and insulin resistance are reported to affect asprosin levels. However, it is not known which physiological signaling or molecular mechanisms balance the homeostasis of asprosin. Our group has shown that asprosin concentrations are significantly higher in obese patients and that body weight changes after bariatric surgery are significantly associated with pre-surgical asprosin levels without associations with blood glucose or cholesterol levels [22]. These results indicated that the regulations of asprosin may not be glucose-dependent since heart failure patients and bariatric surgery outcomes both affected asprosin concentrations. Heart failure patients have multiple neurohormonal abnormalities and disturbed renin-angiotensin axis [26]. Therefore, it is possible that abnormal neurohormonal signaling may also serve as asprosin regulation in addition to the circadian rhythm or fasting status.

Patients with lower or higher asprosin levels exhibited similar serum BNP concentrations. Serum BNP levels are predictors of outcomes in chronic heart failure patients [24]. However, BNP has several limitations for follow-up and diagnosis of heart failure patients [27]. BNP may be affected by renal function, body weight, and lung diseases [28]. BNP measurements alone are not sufficient to guide therapeutic decision-making in patients with heart failure [29]. The fact that patients with different asprosin levels had similar BNP concentrations in our cohort could indicate that the mechanisms linking cardiovascular outcomes in heart failure patients are different from BNP or asprosin.

The data in H9c2 cells showed that asprosin can protect cells from hypoxic injury. This result is consistent with that the higher asprosin concentrations are associated with better outcomes in DCM patients. However, we reasoned that there are still three questions remained to be answered in the future study. First, what is the asprosin receptor in the cardiomyocytes? The previous study has shown that OLFR734 is an asprosin receptor in the liver. Our data showed that *Olfr734* mRNA in the hearts of wild-type mice is significantly lower than the mRNA in the white fat tissues (0.13 ± 0.03 vs. 1 ± 0.05, heart vs. white fat, *P* < 0.05). Moreover, *Olfr734* knockout does not completely abolish the asprosin-stimulated effect on glucose production [21]. These observations suggest that asprosin may have other receptors in different tissues and asprosin may have functions other than glucose homeostasis in cardiomyocytes.

In the H9c2 cardiomyoblast experiments, cells treated with asprosin without hypoxia showed no significant mitochondrial function changes. Instead, cells under hypoxia injury with impaired mitochondrial respiration exhibited a significant recovery of the mitochondrial function after asprosin treatment. This data suggests that asprosin function in H9c2 is hypoxia driven and may only initiate downstream cascades after cells experience hypoxia or ischemic injury. This is similar to the autophagy machinery in the cells. Hypoxia significantly induces autophagy functions in cardiomyocytes and protects cells from hypoxia while cardiomyocytes without hypoxia exhibit minimal autophagy induction [30, 31]. It is not known whether asprosin will activate autophagy flux and future study will be needed.

The function of asprosin is currently unclear except for its glucogenic effects in hepatic cells [18] and orexigenic stimulation in the brain [17]. In failing heart, energy utilization and substrates expenditure are shifting from fatty acid to glucose, lactate, and ketone bodies [32]. This energy substrates switch makes energy production more efficient with 30% more ATP production by carbohydrate oxidation [33]. It is possible that asprosin also has glucogenic effects in cardiomyocytes and provides a beneficial adaptive mechanism for efficient energy production. Further studies will be needed to explore these mechanisms.

## Conclusions

Our clinical studies were performed in a prospective manner with DCM patients in a single medical center. In this DCM cohort study, we did not include a control healthy group. Because our enrollment criteria included the confirmation from cardiac catheterization of normal coronary arteries, we have a smaller number of DCM patient cohort. The small cohort might limit the statistic power and the interpretation of study results. Besides, although we have avoided the possible confounding factors from heterogeneous etiologies and different phenotypes of HF patients with an enrollment of only DCM patients, the findings of our results could not directly apply to patients with other etiologies of heart failure. Future clinical studies with a larger number with a full spectrum of HF patients will be needed. Moreover, we only performed H9c2 cardiomyoblasts studies in vitro to test the mechanisms of asprosin. In vivo animal studies are also required to reconfirm our findings. However, we believe that the DCM cohort provides us with clear clinical observations without the complex confounding factors in heart failure patients of many different etiologies. Moreover, our cardiomyoblast studies in vitro provide direct evidence that asprosin exerts significant effects in the cardiovascular field.

## Data Availability

The data used to support the findings of this study are available from the corresponding author upon request.
